# Non-invasive brain stimulation for treatment-resistant schizophrenia: protocol of a systematic review and network meta-analysis

**DOI:** 10.1186/s13643-024-02585-2

**Published:** 2024-06-24

**Authors:** Spyridon Siafis, Carolin Lorenz, Hui Wu, Yikang Zhu, Johannes Schneider-Thoma, Irene Bighelli, Chunbo Li, Wulf-Peter Hansen, Frank Padberg, Georgia Salanti, Stefan Leucht

**Affiliations:** 1 Technical University of Munich, TUM School of Medicine and Health, Department of Psychiatry and Psychotherapy, Munich, Germany; 2 Shanghai Key Laboratory of Psychotic Disorders, Shanghai Mental Health Centre, Shanghai Jiao Tong University School of Medicine, Shanghai, China; 3 Institute of Psychology and Behavioral Science, Shanghai Jiao Tong University, Shanghai, China; 4 BASTA-Das Bündnis Für Psychisch Erkrankte Menschen, Munich, Germany; 5 Department of Psychiatry and Psychotherapy, LMU University Hospital Munich, Munich, Germany; 6 Institute of Social and Preventive Medicine (ISPM), University of Bern, Bern, Switzerland

**Keywords:** Electroconvulsive therapy, Magnetic seizure therapy, Transcranial magnetic stimulation, Transcranial electric stimulation, Sham intervention, Psychosis, Treatment-resistance, Clinical trial

## Abstract

**Background:**

Non-invasive brain stimulation (NIBS) is a promising intervention for treatment-resistant schizophrenia. However, there are multiple available techniques and a comprehensive synthesis of evidence is lacking. Thus, we will conduct a systematic review and network meta-analysis to investigate the comparative efficacy and safety of NIBS techniques as an add-on to antipsychotics for treatment-resistant schizophrenia.

**Methods:**

We will include single- and double-blind randomized-controlled trials (RCT) comparing any NIBS technique with each other or with a control intervention as an add-on to antipsychotics in adult patients with treatment-resistant schizophrenia. We will exclude studies focusing on predominant negative symptoms, maintenance treatment, and single sessions. The primary outcome will be a change in overall symptoms, and secondary outcomes will be a change in symptom domains, cognitive performance, quality of life, functioning, response, dropouts, and side effects. We will search for eligible studies in previous reviews, multiple electronic databases and clinical trial registries from inception onwards. At least two independent reviewers will perform the study selection, data extraction, and risk of bias assessment. We will measure the treatment differences using standardized mean difference (SMD) and odds ratio (OR) for continuous and dichotomous outcomes, respectively. We will conduct pairwise and network meta-analysis within a frequentist framework using a random-effects model, except for rare event outcomes where we will use a fixed-effects Mantel–Haenszel method. We will investigate potential sources of heterogeneity in subgroup analyses. Reporting bias will be assessed with funnel plots and the Risk of Bias due to Missing Evidence in Network meta-analysis (ROB-MEN) tool. The certainty in the evidence will be evaluated using the Confidence in Network Meta-analysis (CINeMA) approach.

**Discussion:**

Our network meta-analysis would provide an up-to-date synthesis of the evidence from all available RCTs on the comparative efficacy and safety of NIBS for treatment-resistant schizophrenia. This information could guide evidence-based clinical practice and improve the outcomes of patients.

**Systematic review registration:**

PROSPERO-ID CRD42023410645.

**Supplementary Information:**

The online version contains supplementary material available at 10.1186/s13643-024-02585-2.

## Background

Antipsychotic drugs are the mainstay treatment of schizophrenia [1], but a substantial number of patients meet the criteria for treatment-resistant schizophrenia and do not sufficiently respond to antipsychotics [2, 3], including clozapine [4]. In such cases, non-invasive brain stimulation (NIBS) techniques, such as electroconvulsive therapy (ECT), transcranial magnetic stimulation (TMS), and transcranial electric stimulation (tES), can be promising treatment options [5]. However, the evidence regarding the efficacy and safety of NIBS for treatment-resistant schizophrenia is currently inconclusive. Despite this, guidelines recommend considering ECT and TMS due to the high non-response rates to antipsychotics and the lack of other interventions [1, 6].

Existing syntheses of the evidence rely on systematic reviews and meta-analyses with mixed results, that included a few small clinical trials, did not specifically focus on treatment resistance, and applied a pairwise meta-analytic approach (e.g., investigating one pairwise comparison between interventions at a time) [7–16]. Therefore, they provided limited information about the comparative efficacy and safety among the different NIBS techniques and their findings are often difficult to interpret. For example, a Cochrane review found that adjunctive ECT was superior to usual care but not to sham interventions [7].

To overcome these limitations and better inform evidence-based management, we will conduct a network meta-analysis (NMA) to jointly analyze all available clinical trials that investigated NIBS techniques for treatment-resistant schizophrenia.

## Methods/design

The protocol was reported according to the PRISMA statement for protocols [17] (eAppendix 1) and registered on 03.04.2023 with PROSPERO (ID: CRD42023410645) (eAppendix 4). The reporting of the systematic review will follow the PRISMA statement for network meta-analysis [18]. The eAppendix 2 provides information regarding the current status of the review and any changes made from the initial version of the protocol. If further amendments to the protocol are necessary, we will update the PROSPERO registration and include a clear report of any deviations in the published manuscript.

### Eligibility criteria

#### Study design

We will include randomized trials (RCTs) comparing any NIBS to each other or a control condition as an add-on to antipsychotics for treatment-resistant schizophrenia, in which outcome assessors were blinded to the treatment (at least single-blind) [19]. We will exclude maintenance studies, in which patients were stabilized with NIBS before randomization. We will also exclude studies with a high risk of bias in the randomization process [20] and investigate monotherapy treatment or a single session with NIBS. If a trial is described as double-blind, but randomization is not explicitly mentioned, we will assume that the trial was randomized. In case of crossover studies, data from the first crossover phase will be used to avoid carry-over effects [19, 21]. Cluster-randomized trials will be included, and appropriate corrections in the estimations of the relative treatment effects will be applied [22]. There will be no other restrictions in terms of sample size, follow-up time, and country of origin [22].

#### Participants

Adult participants with a treatment-resistant form of schizophrenia, schizoaffective, or schizophreniform disorder will be eligible.

We will accept any study definition of treatment resistance since previous definitions varied widely across trials investigating NIBS [23] and did not fully align with the criteria of the Treatment Response and Resistance in Psychosis (TRRIP) group [2]. The different levels of stringency for defining treatment resistance will be classified as low, intermediate, and high cutoffs, similar to previous reviews [24, 25], and will be examined in subgroup analyses. Accordingly, studies requiring all participants to have treatment-resistant positive symptom domains (e.g., auditory hallucinations) will be eligible, e.g., [26], since positive symptoms have a central role in treatment-resistant schizophrenia [27]. Nevertheless, we will exclude studies in other specific populations, e.g., requiring all participants to have predominant negative symptoms, cognitive impairment, or comorbidities such as depression or drug abuse. In addition, we will assume that patients in the trials received treatment with antipsychotics in cases where it was not explicitly mentioned unless there is explicit information indicating otherwise, as monotherapy treatment with NIBS will be excluded.

There will be no additional restrictions in terms of age (adults-study defined, no upper age limit), setting, gender, ethnicity, severity of illness, and means of diagnosis (operationalized criteria or not). Studies including participants with other mental health conditions would be eligible only if at least 80% of the participants have a diagnosis of schizophrenia, schizoaffective, or schizophreniform disorder.

#### Experimental interventions

Any non-invasive brain stimulation (NIBS) administered as an add-on to antipsychotics will be eligible. There are currently multiple NIBS techniques that could be classified into four general modalities sharing neurophysiological mechanisms:

1. *Electroconvulsive therapy* (ECT) involves the induction of a seizure by administering electrical stimulus with electrodes placed in the scalp, typically under general anesthesia (modified-ECT) [28]. There are different protocols based on the location of electrodes (e.g., bilateral or unilateral, frontal or temporal) and electrical dosage.
2. *Magnetic seizure therapy* (MST) utilizes a magnetic field to induce the seizure and is considered more focal with fewer adverse effects than ECT [29].
3. *Transcranial magnetic stimulation* (TMS) can target distinct brain regions by administering electromagnetic pulses via coils [12, 30]. There are different protocols based on the frequency and pattern of pulses (e.g., repetitive TMS [rTMS] of low or high frequency, priming TMS when the high frequency is followed by low, theta burst stimulation [TBS] when 50 Hz bursts are administered at theta frequency, alpha-synchronized rTMS when stimulation is synchronized to the alpha frequency), focality and depth of stimulation (e.g., deep TMS with H-coils), location of coils (e.g., bilateral or unilateral, and prefrontal, tempoparietal, or cerebellar), and density of sessions (e.g., accelerated when multiple sessions are administered daily in order to condense rTMS within a shorter period of time).
4. *Transcranial electrical stimulation* (tES) involves the administration of weak electrical currents, usually via a bipolar electrode in the scalp [15]. There are different protocols based on the pattern of electrical stimulation (e.g., transcranial direct current stimulation [tDCS], transcranial alternating current stimulation [tACS] with a fixed frequency or transcranial random noise stimulation [tRNS]), and the location of the electrodes (e.g., bilateral or unilateral).

We will exclude NIBS monotherapy and single sessions, as well as other interventions, such as invasive brain stimulation including vagus nerve stimulation and deep brain stimulation, traditional medicine (e.g., acupuncture), psychotherapy, cognitive remediation, and lifestyle interventions. We will also exclude combination treatments such as NIBS combined with psychosocial or pharmacological intervention, except for adjunctive NIBS to treatment as usual with antipsychotic medications (see “Control interventions” section).

#### Control interventions

Any NIBS technique will be compared to each other and to control conditions, which could be classified into three main categories:

1. Sham interventions are procedures that simulate the different NIBS techniques in order to facilitate blinding and control for placebo effects [19]. They should be administered as adjunctive to treatment as usual with antipsychotic medications.
2. Treatment with antipsychotics without sham interventions or NIBS will be considered as treatment as usual (TAU), irrespective of the duration, the number, dose, and type of antipsychotics. Nevertheless, the initiation of a new antipsychotic as an add-on treatment to treatment as usual will not be eligible.
3. Other control conditions (e.g., waiting list) will be eligible if identified during the screening process.

### Outcomes

#### Primary outcome

The primary outcome will be a change in overall symptoms of schizophrenia as measured by the Positive and Negative Syndrome Scale (PANSS) [31], the Brief Psychiatric Rating Scale (BPRS) [32], or any other validated scale [33]. PANSS and BPRS have been used in almost all schizophrenia trials [34], yet some trials investigating NIBS focused on positive symptom domains and did not utilize a score for overall symptoms [11, 16]. Therefore, when scores of overall symptoms will not be available, scores of positive symptoms will be used instead. This decision will allow a comprehensive synthesis of evidence, and will be investigated in a sensitivity analysis (see below “Sensitivity analyses” section).

#### Secondary outcomes

The secondary outcomes will be changes in quality of life, overall functioning, and symptom domains of schizophrenia as measured with validated scales, i.e., positive and negative symptoms, depressive symptoms, and cognitive performance. The cognitive performance will be investigated similarly to our previous analysis [35] and classified into global composite scores and scores for the seven domains of MATRICS [36], including attention/vigilance, speed of processing, working memory, visual learning, verbal learning, reasoning, and problem-solving as well as social cognition.

Moreover, we will examine the number of patients with a positive response to treatment (preferably defined as ≥ 20% reduction of PANSS or BPRS total scores [37], other cut-offs or study definitions will also be eligible), number of participants prematurely discontinued from the studies (i.e., dropouts due to any reason, inefficacy or adverse events), mortality due to any reason, the number of patients with serious adverse events [38], and the number of patients with specific side-effects such as neurological, cognitive, cardiovascular and musculoskeletal [12, 15, 28, 30].

#### Timing of outcome assessment

All outcomes will be assessed at the primary treatment endpoint of each study, which could range from a few days to weeks. There is evidence from antipsychotic trials in schizophrenia, that statistically significant separation between treatment and placebo response could be detected already in the first week, yet clear separation can be observed after at least three weeks [39]. However, the time course of treatment response with NIBS is unclear and can also vary across modalities as some studies demonstrated the efficacy of tES and TMS after five days of treatment [26, 40]. Therefore, we will accept any treatment endpoint (except after a single session) and we will extract data at all available time points for the primary outcome and at the treatment endpoint for the secondary outcomes. Given the potential relationship between treatment duration and the number of sessions, as well as their variation across different modalities and potential influence from other characteristics, we will attempt to a posteriori group the treatment duration and number of sessions of the treatments into meaningful categories to examine their impact on the primary outcome (see “Subgroup analyses” section).

Further, we will evaluate the primary outcome at several follow-up timepoints after the end of treatment (1, 3, 6, and 12 months) as secondary outcomes, since there is mixed evidence on the maintenance of any improvement after the end of treatment, e.g., [26, 41].

### Information sources and search strategy

We will search multiple electronic databases without restrictions in terms of document type, publication status, publication period or language [22], i.e., EMBASE, PubMed, MEDLINE, PsycINFO, the clinical trials registers of the Cochrane Central Register of Controlled Trials (CENTRAL), ClinicalTrials.gov and WHO International Clinical Trials Registry Platform, and the three main Chinese databases of Wanfang Database, China National Knowledge Infrastructure (CNKI), and China Biology Medicine disc. The search terms will be developed together with experienced information specialists and methodologists (FS, JX). The search strategies on PubMed and CNKI are presented in eAppendix 3. We will also inspect reference lists of all included studies and previous reviews investigating NIBS for schizophrenia, e.g., [7–12, 14–16, 42]. In case of missing information, we will contact the first and/or corresponding author of included studies published in the last 30 years and companies manufacturing NIBS devices.

### Study selection and data collection

#### Study selection

Two independent reviewers will screen identified titles/abstracts for inclusion, and disagreements will be resolved by discussion or by acquiring full articles for further inspection. Full texts of relevant titles/abstracts will be obtained, and in a second step, two independent reviewers will evaluate them against the eligibility criteria. Study selection will be reported in flow diagrams according to the PRISMA statement [18, 43] and we will provide a table listing the studies excluded at the full-text level, along with the respective reasons for exclusion. Disagreements will be resolved by discussion with a third senior reviewer, or contacting study authors. Records will be managed using Citavi [44].

#### Data extraction

Two independent reviewers will extract data on specifically developed forms in a Microsoft Access database that is tailor-made by our group for schizophrenia trials. Discrepancies in double data extraction will be identified by an algorithm, and doubts will be resolved by discussion with a third senior reviewer or by contacting study authors. We will extract information about study design and methodology, participant and intervention characteristics, and outcome measures. We will provide a table of study characteristics for the included studies.

For continuous outcomes, we will prefer change over endpoint scores, and methods accounting for missing outcome data (e.g., mixed-models of repeated measurement (MMRM), multiple imputations) over last-observation carried forward (LOCF) and over observed cases. Missing standard deviations (SD) will be derived from test statistics [22], by contacting study authors, or from SDs of other included studies using a validated imputation method [45].

For dichotomous outcomes, in case studies present only observed cases, we will assume that participants lost to follow-up had not responded to treatment or had not developed side effects. We judge this as appropriate since otherwise many rare side-effects would be overestimated. It is also a conservative approach regarding efficacy outcomes.

#### Risk of *bias* assessment

Two independent reviewers will evaluate the risk of bias of the primary outcome and dropouts due to any reason using the risk of bias tool RoB-2 [20], which considers the domains of the randomization process, deviations of indented interventions, missing outcome data, measurement of the outcome and selection of the reported result. Within-study reporting bias will additionally be evaluated with the Risk Of Bias due to Missing Evidence in Network meta-analysis (RoB-MEN) tool [46] (see below “Small-study effects and reporting bias” section). Discrepancies will be resolved by discussion with a third senior reviewer or by contacting study authors.

### Data synthesis

#### Two-step procedure

We plan to conduct a network meta-analysis on the comparative efficacy and safety of NIBS. Network meta-analysis can combine direct and indirect evidence, simultaneously analyze all available clinical trials with increased precision and power, compare all interventions to each other, and provide treatment rankings [22].

We will follow a two-step procedure. First, we will perform a series of pairwise meta-analyses by investigating RCTs that compared directly two interventions. Second, if the requirements of NMA are met, we will conduct NMA in a frequentist framework [47]. We will use a random-effects model since heterogeneity is likely [48], and a fixed-effects Mantel–Haenszel method in case of rare dichotomous outcomes [49], such as mortality [50].

#### Effect-sizes

The effect size for continuous outcomes will be a standardized mean difference (SMD) since different rating scales are expected, and for dichotomous outcomes will be odds ratio (OR) because of their preferred mathematical properties [51, 52]. Effect sizes will be presented with their 95% confidence intervals. If heterogeneity is not large and treatment effects are estimated with comparable uncertainty, treatments will be ranked in the network meta-analysis using *P*-scores, the frequentist analog of the surface under the cumulative ranking curve (SUCRA) [53].

#### Network geometry

**Fig. 1:**
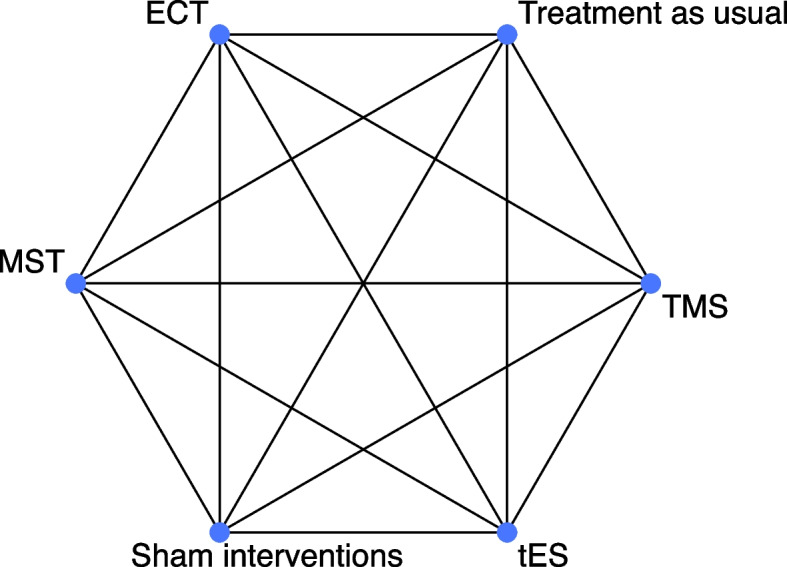
Theoretical network diagram for the primary analysis. The nodes represent the different interventions, and the edges connecting the different nodes represent the number of studies that compared directly two interventions. Their size will be proportionate to the number of participants and number of studies available, respectively

The network geometry will be presented with a network plot, in which nodes will represent different interventions and edges between nodes will represent the available trials that investigated a direct comparison between interventions. In the primary analysis, we will define nodes according to general modalities of NIBS, i.e., ECT, MST, TMS, and tES, and control conditions, i.e., sham interventions (reference comparator) and treatment as usual (Fig. 1). Thus, different NIBS protocols of the same modality and sham interventions will be merged. This decision is of clinical relevance due to the weak conceptual foundation of the majority of the protocols, and statistical power will be increased. Nevertheless, we will conduct sensitivity analyses by applying different definitions of nodes for NIBS at the level of a specific protocol, by defining distinct nodes for the sham interventions of the different modalities (e.g., sham-ECT, sham-TMS), and by classifying sham interventions into active (such as weak stimulation via coils applied with an angle of 45° or 90°) and inactive (such as inactive coils not producing magnetic fields) [54].

#### Transitivity assumption

We will include only trials in patients with treatment-resistant schizophrenia and exclude those in specific populations, and therefore, we will assume that patients in eligible trials are equally likely to be randomized to any of the interventions (i.e., transitivity assumption). The transitivity assumption is required for valid indirect comparisons and will be further explored by examining the distribution of potential effect modifiers across treatment comparisons [22], such as age, sex, baseline severity of symptoms, duration of illness, definition of treatment-resistance [24, 25], diagnosis (schizophrenia or schizoaffective disorder), rating scale, dose and type of antipsychotics, treatment duration and frequency of NIBS, blinding status, publication year, sample size, and sponsorship.

#### Assessment of heterogeneity

A common between-study variance (τ^2^) will be assumed across treatment comparisons within a network [22]. Heterogeneity will be quantified by comparing the τ^2^ with its empirical distributions [55, 56] and the magnitude will be classified into low, moderate, and high.

#### Assessment of incoherence

The statistical agreement between direct and indirect evidence will be evaluated within closed loops with the separating indirect from direct evidence approach (SIDE) and in the entire network with a design-by-treatment interaction test [22]. Tests of incoherence have low statistical power, and thus, sources of incoherence will be explored even in the absence of a statistically significant test result. In the presence of incoherence, we will explore analytical strategies, such as splitting the network into subgroups or using Bayesian network meta-regression to explore sources of incoherence and heterogeneity [22] (see below “Subgroup analyses” section).

#### Subgroup analyses

We will investigate potential sources of heterogeneity and/or incoherence in the primary outcome with subgroup analyses on (a) baseline severity of overall symptoms, (b) definition of treatment-resistance [24, 25], (c) duration of illness, (d) publication year, (e) sample size, (f) treatment duration, and (g) number of sessions. If network meta-regression is deemed appropriate and feasible for examining these potential effect modifiers, we will fit regressions in a Bayesian setting and assess the influence of modifiers by examining the credible intervals of the regression coefficients and evaluating changes in both heterogeneity and inconsistency between the unadjusted and adjusted models.

#### Sensitivity analyses

The robustness of the results for the primary outcome will be investigated with sensitivity analysis by excluding studies (a) that were only single-blind, (b) with an overall high risk of bias, (c) with implied randomization, (d) that did not use operationalized diagnostic criteria, (e) in which patients were assumed to receive antipsychotics if it was not clearly written in the study, (f) that required all patients to have treatment-resistant positive symptom domains, (g) with rating scales of positive symptoms used for the primary outcome, (h) imputed values, and (i) from mainland China [57], as well as by defining (j) different nodes for active and inactive sham interventions, (k) distinct nodes for sham interventions of different modalities and (l) different nodes for specific NIBS protocols.

#### Small-study effects and reporting *bias*

We will aim to include both published and unpublished RCTs. Small-study effects and the possible publication bias will be examined for the primary outcome (i.e., overall symptoms) and dropouts due to any reason with contour-enhanced funnel plots for pairwise meta-analysis when more than 10 studies are available [22], and comparison-adjusted funnel plots assuming the direction of bias towards newer interventions [58]. We will further evaluate reporting bias for the entire networks using the RoB-MEN tool [46], which considers within- and across-study assessments of reporting bias (see “Risk of bias assessment” section).

#### Confidence in the evidence

The confidence in the evidence will be evaluated for the primary outcome (i.e., overall symptoms) and dropouts due to any reason using the Confidence in Network Meta-Analysis (CINeMA) approach [59], considering within-study bias, reporting bias, indirectness, impression, heterogeneity, and incoherence.

Data analysis will be conducted in R statistical software [60] using the packages meta [61] and netmeta [62]. Bayesian network meta-regressions will be conducted with self-programmed routines in JAGS [63].

## Discussion

Non-response to antipsychotics and treatment-resistance is a frequent condition in patients with schizophrenia [2], and it is associated with a poorer prognosis [64], reduced quality of life [65], and higher costs [65]. Among the limited number of interventions, non-invasive brain stimulation (NIBS) namely ECT and TMS, are recommended by current guidelines [1, 6], although the evidence is yet inconclusive and based on scattered pairwise meta-analyses, e.g., [7–16]. Our planned network meta-analysis would jointly synthesize the evidence from all available RCTs on the comparative efficacy and safety of NIBS techniques as an add-on to antipsychotics for treatment-resistant schizophrenia. Thus, this information could inform treatment guidelines and clinical practice [66].

There are potential challenges and limitations.

- First, our network meta-analysis focuses on the acute phase trials investigating NIBS for treatment-resistance schizophrenia, and other potentially important aspects will not be considered, such as trials investigating maintenance treatment [67], predominant negative symptoms [68], and cognitive impairments [69]. However, we will explore these factors as secondary outcomes, specifically examining the different symptom domains of schizophrenia and the maintenance of symptom improvement following the completion of the treatment. Moreover, other intervention modalities that can be useful for treatment-resistant schizophrenia will not be considered, e.g., augmentation with another medication or psychological treatments [70].
- Second, we will search Chinese databases and include studies from mainland China. Chinese databases represent a unique database for this research question and should not be disregarded, given the large number of available trials, and that ECT is widely used for schizophrenia in contrast to other countries [71]. However, studies from mainland China are often excluded from systematic reviews due to inadequate reporting and inappropriate randomization [57, 72]. Therefore, they will be carefully evaluated by native Chinese researchers and excluded in a sensitivity analysis.
- Third, we will aim to analyze the safety of NIBS by considering dropouts due to adverse events, serious adverse events, and specific side effects. However, NIBS techniques can have distinct side-effect profiles, and thus, a long list of diverse side-effects is expected. In addition to this, the inconsistent reporting and assessment of side effects in clinical trials [73] may not allow a network meta-analysis for some outcomes, and in that case, a pairwise meta-analysis will be presented.
- Last, it can be expected according to previous systematic reviews [7–16, 68] that there will be a few studies for comparison and that networks will be mainly star-shaped. In that case, heterogeneity and incoherence could not be well evaluated, and thus, we will downgrade accordingly the evidence in CINeMA and carefully interpret the findings.

In conclusion, our network meta-analysis could provide up-to-date information about the comparative efficacy and safety of non-invasive brain stimulation for treatment-resistant schizophrenia, which could guide evidence-based clinical practice and improve the outcomes of patients.

### Supplementary Information

Additional file 1: eAppendix 1. PRISMA-P. eAppendix 2. Current status and modifications from the first version of the PROSPERO registration. eAppendix 3. Search strategies. eAppendix 4. PROSPERO Registration CRD42023410645.

## Data Availability

Not applicable.

## Abbreviations

BPRS: Brief Psychiatric Rating Scale
CENTRAL: Cochrane Central Register of Controlled Trials
CINeMA: Confidence in Network Meta-Analysis
CNKI: China National Knowledge Infrastructure
CSG: Cochrane Schizophreni Group
ECT: Electroconvulsive therapy
LOCF: Last-observation carried forward
MCCB: MATRICS Consensus Cognitive Battery
MMRM: Mixed-models of repeated measurement
MST: Magnetic seizure therapy
NIBS: Non-invasive brain stimulation
NMA: Network meta-analysis
OR: Odds ratio
PANSS: Positive and Negative Syndrome Scale
RCT: Randomized-controlled trials
ROB-MEN: Risk Of Bias due to Missing Evidence in Network meta-analysis
rTMS: Repetitive transcranial magnetic stimulation
SD: Standard deviations
SIDE: Separating indirect from direct evidence approach
SMD: Standardized mean difference
SUCRA: Surface under the cumulative ranking curve
tACS: Transcranial alternating current stimulation
TAU: Treatment as usual
TBS: Theta burst stimulation
tDCS: Transcranial direct current stimulation
tES: Transcranial electric stimulation
TMS: Transcranial magnetic stimulation
TRRIP: Treatment Response and Resistance in Psychosis
