# Supplementary material for: Non-invasive brain stimulation for treatment-resistant schizophrenia: protocol of a systematic review and network meta-analysis
Source: Syst Rev. 2024 Jun 24;13:165. doi: 10.1186/s13643-024-02585-2 (PMC11195004; doi:10.1186/s13643-024-02585-2)
Supplement: Supplementary file 1 — Additional file 1: eAppendix 1. PRISMA-P. eAppendix 2. Current status and modifications from the first version of the PROSPERO registration. eAppendix 3. Search strategies. eAppendix 4. PROSPERO Registration CRD42023410645. [file 13643_2024_2585_MOESM1_ESM.pdf]

Non-invasive brain stimulation for treatment-resistant schizophrenia: protocol of a systematic review and network meta-analysis

Table of Contents

eAppendix-1: PRISMA-P *2*

eAppendix-2: Current status and modifications from the first version of the PROSPERO registration *4*

- Current status of the review 4
- Modifications from the first version of the PROSPERO protocol 4

eAppendix-3: Search strategies *6*

- Search strategy in PubMed (excluding MEDLINE that will be searched via Ovid): 6
- Search strategy in CNKI: 7

eAppendix-4: PROSPERO registration CRD42023410645 ..... *8*

eAppendix-1: PRISMA-P

PRISMA-P (Preferred Reporting Items for Systematic review and Meta-Analysis Protocols) 2015 checklist: recommended items to address in a systematic review protocol*

Section and topic	Item No	Checklist item	Page
**ADMINISTRATIVE INFORMATION**			
Title:			
Identification	1a	Identify the report as a protocol of a systematic review	1
Update	1b	If the protocol is for an update of a previous systematic review, identify as such	Not an update
Registration	2	If registered, provide the name of the registry (such as PROSPERO) and registration number	3, 5
Authors:			
Contact	3a	Provide name, institutional affiliation, e-mail address of all protocol authors; provide physical mailing address of corresponding author	1
Contributions	3b	Describe contributions of protocol authors and identify the guarantor of the review	20
Amendments	4	If the protocol represents an amendment of a previously completed or published protocol, identify as such and list changes; otherwise, state plan for documenting important protocol amendments	5, eAppendix-2
Support:			
Sources	5a	Indicate sources of financial or other support for the review	20
Sponsor	5b	Provide name for the review funder and/or sponsor	20
Role of sponsor or funder	5c	Describe roles of funder(s), sponsor(s), and/or institution(s), if any, in developing the protocol	20-21
**INTRODUCTION**			
Rationale	6	Describe the rationale for the review in the context of what is already known	4
Objectives	7	Provide an explicit statement of the question(s) the review will address with reference to participants, interventions, comparators, and outcomes (PICO)	4
**METHODS**			
Eligibility criteria	8	Specify the study characteristics (such as PICO, study design, setting, time frame) and report characteristics (such as years considered, language, publication status) to be used as criteria for eligibility for the review	5-9
Information sources	9	Describe all intended information sources (such as electronic databases, contact with study authors, trial registers or other grey literature sources) with planned dates of coverage	10-11
Search strategy	10	Present draft of search strategy to be used for at least one electronic database, including planned limits, such that it could be repeated	eAppendix-2
Study records:			
Data management	11a	Describe the mechanism(s) that will be used to manage records and data throughout the review	11
Selection process	11b	State the process that will be used for selecting studies (such as two independent reviewers) through each phase of the review (that is, screening, eligibility and inclusion in meta-analysis)	11
Data collection process	11c	Describe planned method of extracting data from reports (such as piloting forms, done independently, in duplicate), any processes for obtaining and confirming data from investigators	11-12
Data items	12	List and define all variables for which data will be sought (such as PICO items, funding sources), any pre-planned data assumptions and simplifications	8-12
Outcomes and prioritization	13	List and define all outcomes for which data will be sought, including prioritization of main and additional outcomes, with rationale	8-11
Risk of bias in individual studies	14	Describe anticipated methods for assessing risk of bias of individual studies, including whether this will be done at the outcome or study level, or both; state how this information will be used in data synthesis	12

Data synthesis	15a	Describe criteria under which study data will be quantitatively synthesised	12
	15b	If data are appropriate for quantitative synthesis, describe planned summary measures, methods of handling data and methods of combining data from studies, including any planned exploration of consistency (such as I^2, Kendall's τ)	12-14
	15c	Describe any proposed additional analyses (such as sensitivity or subgroup analyses, meta-regression)	15
	15d	If quantitative synthesis is not appropriate, describe the type of summary planned	Not applicable
Meta-bias(es)	16	Specify any planned assessment of meta-bias(es) (such as publication bias across studies, selective reporting within studies)	15-16
Confidence in cumulative evidence	17	Describe how the strength of the body of evidence will be assessed (such as GRADE)	16

*** It is strongly recommended that this checklist be read in conjunction with the PRISMA-P Explanation and Elaboration (cite when available) for important clarification on the items. Amendments to a review protocol should be tracked and dated. The copyright for PRISMA-P (including checklist) is held by the PRISMA-P Group and is distributed under a Creative Commons Attribution Licence 4.0.**

From: Shamseer L, Moher D, Clarke M, Ghersi D, Liberati A, Petticrew M, Shekelle P, Stewart L, PRISMA-P Group. Preferred reporting items for systematic review and meta-analysis protocols (PRISMA-P) 2015: elaboration and explanation. BMJ. 2015 Jan 2;349(jan02 1):g7647.

eAppendix-2: Current status and modifications from the first version of the PROSPERO registration

Current status of the review

At the time of registering the protocol in PROSPERO, we had initiated preliminary searches and piloted the study selection process, but we had not yet completed them. We had not started the study selection and data extraction.

At the time of submitting the current protocol for publication, we have conducted the final search for studies, and we are currently in the process of conducting the study selection (which is not yet completed) and piloting the data extraction forms (the final data extraction stage has not yet started).

Modifications from the first version of the PROSPERO protocol

The initial PROSPERO registration on 03.04.2023 is available in eAppendix-4. There were no major differences between the current version of the protocol and the initial PROSPERO registration. A few changes are described below:

1. We described some parts in more detail given the word count limitations of the PROSPERO registration such as the consideration of a network meta-regression in a Bayesian setting if it is deemed appropriate and feasible. This had been already planned in the original grant proposal of the project.
2. We will investigate the number of sessions as potential effect-modifiers. Given the potential relationship between treatment duration and the number of sessions, as well as their variation across different modalities and potential influence from other characteristics, we will attempt to *a posteriori* group the treatment duration and number of sessions of the treatments into meaningful categories to examine their impact on the primary outcome (see "Subgroup analyses").

3. We will also investigate duration of illness as potential effect-modifier. This variable was already mentioned in the grant proposal of the project.
4. In the PROSPERO registration it was stated that we will conduct subgroup analyses to investigate differences in the response across sham interventions and other control groups. In the current version of the protocol, we revised this section by stating that we will conduct a sensitivity analysis by conducting network meta-analysis and defining distinct nodes for sham interventions from different modalities. This would be a more appropriate method to investigate differences across the sham interventions of the different modalities.

eAppendix-3: Search strategies

Search strategy in PubMed (excluding MEDLINE that will be searched via Ovid):

("Schizophrenia Spectrum and Other Psychotic Disorders"[MH] OR Schizophrenia[MH] OR Psychotic Disorders[MH:NoExp] OR Schizotypal Personality Disorder[MH] OR Schizophreni*[TIAB] OR Schizoaffective[TIAB] OR Schizo-Affective[TIAB] OR Schizotyp*[TIAB] OR Psychotic*[TIAB] OR Psychosis[TIAB] OR Psychoses[TIAB] OR Chronic Psychiatric Illness*[TIAB] OR Chronic Psychiatric Disorder*[TIAB] OR Chronic Mental Illness*[TIAB] OR Chronic Mental Disorder*[TIAB] OR Severe Psychiatric Illness*[TIAB] OR Severe Psychiatric Disorder*[TIAB] OR Severe Mental Illness*[TIAB] OR Severe Mental Disorder*[TIAB] OR Serious Psychiatric Illness*[TIAB] OR Serious Psychiatric Disorder*[TIAB] OR Serious Mental Illness*[TIAB] OR Serious Mental Disorder*[TIAB]) AND (Convulsive Therapy[MH:NoExp] OR Electroconvulsive Therapy[MH] OR Electric Stimulation Therapy[MH:NoExp] OR Transcranial Direct Current Stimulation[MH] OR Electroshock[MH] OR Magnetic Field Therapy[MH] OR Transcranial Magnetic Stimulation[MH] OR Deep Brain Stimulation[MH] OR Brain Depth Stimulation*[TIAB] OR Brain Stimulation*[TIAB] OR Convulsive Therap*[TIAB] OR Current Stimulation[TIAB] OR Electric Convuls*[TIAB] OR Electric Field Stimulation*[TIAB] OR Electric Stimulation*[TIAB] OR Electrical Stimulation*[TIAB] OR Electro Stimulation*[TIAB] OR Electroconvuls*[TIAB] OR Electromagnetic Therap*[TIAB] OR Electroshock*[TIAB] OR Electrostimul*[TIAB] OR Electrotherap*[TIAB] OR Magnetic Field Therap*[TIAB] OR Magnetic Seizure*[TIAB] OR Magnetic Stimulation*[TIAB] OR Magnetic Therap*[TIAB] OR Magnetotherap*[TIAB] OR Random Noise Stimulation*[TIAB] OR Shock*[TIAB] OR Theta Burst Stimulation*[TIAB] OR TMS[TIAB] OR aTMS[TIAB] OR dTMS[TIAB] OR pTMS[TIAB] OR rTMS[TIAB] OR sTMS[TIAB] OR tRNS[TIAB] OR tDCS[TIAB] OR tACS[TIAB] OR ECT[TIAB] OR tES[TIAB] OR TBS[TIAB] OR cTBS[TIAB] OR iTBS[TIAB] OR LCE[TIAB] OR MST[TIAB] OR ECS Therap*[TIAB]) AND (Randomized Controlled Trial[PT] OR Controlled Clinical Trial[PT] OR Pragmatic Clinical Trial[PT] OR Randomized[TIAB] OR Randomised[TIAB] OR Placebo[TIAB] OR Randomly[TIAB] OR Trial[TIAB] OR Groups[TIAB]) NOT MEDLINE[SB]

Search strategy in CNKI:

(SU%=精神分裂症+精神分裂+偏执型障碍+分裂型障碍+分裂型精神障碍+分裂情感性障碍+精神病+精神疾病 OR TKA=精神分裂症+精神分裂+偏执型障碍+分裂型障碍+分裂型精神障碍+分裂情感性障碍+精神病+精神疾病) AND (SU%=侵入性脑刺激+ NIBS+非侵入脑刺激+无创脑刺激+无创性脑刺激+非侵入性神经刺激+非侵入性神经调节+无创神经调节+经颅磁刺激+ Rtms+ aTMS+ pTMS+ sTMS+ dTMS+经颅直流电刺激+经颅电刺激+tDCS+经颅交流电刺激+经颅随机电刺激+tACS+经颅微电流刺激+经颅超声刺激+电刺激+磁疗+电磁疗法+磁刺激+磁休克+磁痉挛+磁抽搐+电休克+电惊厥+惊厥疗法+电抽搐+MECT+低电荷电疗+低电荷电疗法+低电量治疗+低电量电抽搐+经颅随机噪声+经颅随机噪音+tRNS+经颅磁治疗+ Theta 节律刺激+Theta 波刺激+ theta 脉冲刺激+iTBS+ cTBS+惊厥疗法+模式化刺激+θ短阵快速脉冲刺激+θ突发刺激+θ脉冲刺激+爆发式磁刺激+爆发式刺激+θ爆发刺激 OR TKA=侵入性脑刺激+ NIBS+非侵入脑刺激+无创脑刺激+无创性脑刺激+非侵入性神经刺激+非侵入性神经调节+无创神经调节+经颅磁刺激+ Rtms+ aTMS+ pTMS+ sTMS+ dTMS+经颅直流电刺激+经颅电刺激+tDCS+经颅交流电刺激+经颅随机电刺激+tACS+经颅微电流刺激+经颅超声刺激+电刺激+磁疗+电磁疗法+磁刺激+磁休克+磁痉挛+磁抽搐+电休克+电惊厥+惊厥疗法+电抽搐+MECT+低电荷电疗+低电荷电疗法+低电量治疗+低电量电抽搐+经颅随机噪声+经颅随机噪音+ tRNS+经颅磁治疗+ Theta 节律刺激+Theta 波刺激+ theta 脉冲刺激+iTBS+ cTBS+惊厥疗法+模式化刺激+θ短阵快速脉冲刺激+θ突发刺激+θ脉冲刺激+爆发式磁刺激+爆发式刺激+θ爆发刺激) AND (SU%=随机+盲法+双盲+单盲+三盲+交叉+RCT OR TKA=随机+盲法+双盲+单盲+三盲+交叉+RCT)

Non-invasive brain stimulation for treatment-resistant schizophrenia: protocol of a systematic review and network meta-analysis

To enable PROSPERO to focus on COVID-19 submissions, this registration record has undergone basic automated checks for eligibility and is published exactly as submitted. PROSPERO has never provided peer review, and usual checking by the PROSPERO team does not endorse content. Therefore, automatically published records should be treated as any other PROSPERO registration. Further detail is provided here.

Citation

Carolin Lorenz, Spyridon Siafis, Hui Wu, Johannes Schneider-Thoma, Irene Bighelli, Yikang Zhu, Chunbo Li, Wulf-Peter Hansen, Frank Padberg, Georgia Salanti, Stefan Leucht. Non-invasive brain stimulation for treatment-resistant schizophrenia: protocol of a systematic review and network meta-analysis. PROSPERO 2023 CRD42023410645 Available from: https://www.crd.york.ac.uk/prospero/display_record.php?ID=CRD42023410645

Review question

Are different non-invasive brain stimulation (NIBS) techniques effective and safe for people with treatment-resistant schizophrenia?

Searches

We will search multiple electronic databases without restrictions in terms of document type, publication status, publication period or language (Higgins et al 2019), i.e., the registry of the Cochrane Schizophrenia Group (CSG) (Shokraneh et al 2020), EMBASE, PubMed, MEDLINE, PsycINFO, the clinical trials registers of the Cochrane Central Register of Controlled Trials (CENTRAL), ClinicalTrials.gov and WHO International Clinical Trials Registry Platform, and the four main Chinese databases of Chongqing VIP Database, Wanfang Database, China National Knowledge Infrastructure, and China Biology Medicine disc. The search terms will be developed together with experienced information specialists (FS, JX). We will also inspect reference lists of all included studies and previous reviews investigating NIBS for schizophrenia, e.g. (Sinclair et al 2019, Wang et al 2018, Lefaucheur et al 2020, Cheng et al 2020). In case of missing information, we will contact the first and/or corresponding author of included studies published in the last 30 years and companies manufacturing NIBS devices.

Types of study to be included

We will include randomised trials (RCTs) comparing any NIBS to each other or a control condition for treatment-resistant schizophrenia, in which outcome assessors were blinded to the treatment (at least single-blind) (Brunoni et al 2011). We will exclude maintenance studies, in which patients were stabilized with NIBS before randomization. We also will exclude studies with a high risk for bias in the randomization process (Sterne et al 2019). If a trial is described as double-blind, but randomization is not explicitly mentioned, we will assume that the trial was randomized. In case of crossover studies, data from first crossover phase will be used to avoid carry-over effects (Elbourne et al 2002). Cluster-randomized trials will be included, and appropriate corrections will be applied (Higgins et al 2019). There will be no other restriction in terms of sample size, follow-up time and country of origin.

Condition or domain being studied

Schizophrenia

Participants/population

Adult participants with a treatment-resistant form of schizophrenia or schizophrenia-like disorder will be eligible (at least 80% of the participants in a trial). We will accept any study definition of treatment-resistance because previous definitions varied widely across trials investigating NIBS (Kronick et al 2021) and did not fully align with the criteria of the Treatment Response and Resistance in Psychosis (TRRIP) group (Howes et al 2017). Accordingly, studies that required all participants to have treatment-resistant positive symptom domains (e.g., auditory hallucinations) will be eligible, since positive symptoms have a central role in treatment-resistant schizophrenia (Lee et al 2015). Nevertheless, we will exclude studies in other specific populations, e.g., requiring all participants having predominant negative symptoms, cognitive impairment, or comorbidities such as depression or drug abuse. In addition, we will assume that patients in the trials received treatment with antipsychotics, if not explicitly mentioned but not otherwise reported, since antipsychotics are the mainstay treatment (Hasan et al 2012). There will be no additional restriction in terms of age (adults-study defined, no upper age limit), setting, gender, ethnicity, severity of illness, and means of diagnosis (operationalized criteria or not).

Intervention(s), exposure(s)

Any NIBS administered as adjunctive to antipsychotics will be eligible.

1. Electroconvulsive therapy (ECT) involves the induction of a seizure by administering electrical stimulus with electrodes placed in the scalp (modified-ECT) (Andrade et al 2016).

2. Magnetic seizure therapy (MST) utilizes a magnetic field to induce the seizure, and is considered more focal with fewer adverse effects than ECT (Lisanby et al 2003).

3. Transcranial magnetic stimulation (TMS) can target distinct brain regions by administering electromagnetic pulses via coils (Lefaucheur et al 2020). There are different protocols based on the frequency and pattern of pulses (e.g. repetitive TMS of low or high frequency, priming TMS, theta burst stimulation, alpha-synchronized rTMS, focality and depth of stimulation, location of coils, and density of sessions

4. Transcranial electrical stimulation (tES) involves the administration of weak electrical currents, usually via a bipolar electrode in the scalp (Fregni et al 2021). There are different protocols based on the pattern of electrical stimulation

We will exclude NIBS monotherapy and single sessions, as well as invasive brain stimulation (e.g., vagus nerve stimulation, deep brain stimulation), traditional medicine (e.g., acupuncture), psychotherapy, cognitive remediation, lifestyle interventions and combination treatments (e.g., NIBS combined with an antipsychotic or another pharmacological intervention initiated during the RCT).

Comparator(s)/control

Any NIBS technique will be compared with each other and with control conditions, which could be classified into three main categories:

1. Sham interventions are procedures that simulate the different NIBS techniques in order to facilitate blinding and control for placebo effects. They should be administered as adjunctive to antipsychotic medications.

2. Treatment as usual (TAU) will be considered the treatment with antipsychotics without sham intervention or NIBS, irrespective of the duration, the number, dose and type of antipsychotics. Nevertheless, treatment with antipsychotics that was initiated during the RCT will be excluded.

3. Other control conditions (e.g., waiting list) will be eligible if identified during the screening process.

Context

There are no restrictions in terms of setting, for example,

Main outcome(s)

The primary outcome will be change in overall symptoms of schizophrenia as measured by Positive and Negative Syndrome Scale (PANSS) (Kay et al 1987), the Brief Psychiatric Rating Scale (BPRS) (Overall et al 1962) or any other validated scale (Marshall et al 2000). PANSS and BPRS have been used in almost all schizophrenia trials (Huhn et al 2020), yet some trials investigating NIBS focused on positive symptom domains and did not utilize a score for overall symptoms (Cheng et al 2020). Therefore, when scores of overall symptoms will not be available, scores of positive symptoms will be used.

Measures of effect

The effect-size for continuous outcomes will be standardized mean difference (SMD), since different rating scales are expected, and for dichotomous outcomes will be odds ratio (OR) because of their preferred mathematical properties (Doi et al 2020). Effect sizes will be presented with their 95% confidence intervals. Treatments will be ranked in the network meta-analysis using P-scores, the frequentist analogue of the surface under the cumulative ranking curve (SUCRA) (Rücker et al 2015).

For continuous outcomes, we will prefer change over endpoint scores, and methods accounting for missing outcome data (e.g., mixed-models of repeated measurement (MMRM), multiple imputation, last-observation carried forward (LOCF)) over observed cases. Missing standard deviations (SD) will be derived from test statistics (Higgins et al 2019), by contacting study authors, or from SDs of other included studies using a validated imputation method (Furukawa et al 2006).

For dichotomous outcomes, we will follow an intention-to-treat (ITT), and in case studies present only observed cases, we will assume that participants lost to follow-up had not responded to treatment or not developed side-effects.

Additional outcome(s)

The secondary outcomes will be change in quality of life, overall functioning and symptom domains of schizophrenia as measured with validated scales, i.e., positive and negative symptoms, depressive symptoms and cognitive performance. Cognitive performance will be classified into global composite scores and scores for the seven domains of MATRICS (Nuechterlein et al 2008), i.e., attention/vigilance, speed of processing, working memory, visual learning, verbal learning, reasoning and problem solving, and social cognition.

We will also examine the number of patients with a positive response to treatment (preferably defined as at least 20% reduction of PANSS or BPRS total scores (Leucht et al 2005), other cut-offs or study definitions will also be eligible), number of participants prematurely discontinued from the studies (i.e., dropouts due to any reason, inefficacy or adverse events), mortality due to any reason, the number of patients with serious adverse events, and the number of patients with specific side-effects, e.g., neurological, cognitive, cardiovascular, musculoskeletal.

Measures of effect

See measures of effect under point 24.

Data extraction (selection and coding)

Study selection: Two independent reviewers will screen identified title/abstracts for inclusion, and disagreements will be resolved by discussion or by acquiring full articles for further inspection. Full-texts of relevant title/abstract will be obtained, and in a second step, two independent reviewers will evaluate them against the eligibility criteria. Disagreements will be resolved by discussion with a third senior reviewer, or contacting study authors. Records will be managed using Ryanair and Citavi®.

Data extraction: Two independent reviewers will extract data on specifically developed forms in a Microsoft Access® database that is tailor-made by our group for schizophrenia trials. Discrepancies in double data extraction will be identified by an algorithm, and doubts will be resolved by discussion with a third senior reviewer or by contacting study authors. We will extract information about study design and methodology, participant and intervention characteristics, and outcome measures.

Risk of bias (quality) assessment

Two independent reviewers will evaluate the risk of bias for the primary outcome (i.e., overall symptoms) and dropouts due to any reason using the Cochrane risk-of-bias tool 2 (Sterne et al 2019), which considers the domains of randomization process, deviations of indented interventions, missing outcome data, measurement of the outcome and selection of the reported result. Within-study reporting bias will additionally be evaluated with the Risk of Bias due to Missing Evidence in Network meta-analysis (RoB-MEN) tool (Chiocchia et al 2021). Discrepancies will be resolved by discussion with third senior reviewer or by contacting study authors.

Strategy for data synthesis

We will follow a two-step procedure. First, we will perform pairwise meta-analyses by investigating RCTs that compared directly two interventions. In a second step, if the requirements of NMA are met, we will conduct NMA in a frequentist framework using graph-theoretical methods (Rücker et al 2012). We will use a random-effects model, and a fixed-effects Mantel-Haenszel method in case of rare dichotomous outcomes.

The network geometry will be presented with a network plot, in which nodes will represent different interventions and edges between nodes will represent the available trials that investigated a direct comparison between interventions.

We will restrict to trials in treatment-resistant schizophrenia and exclude those in specific populations, and therefore, we will assume that patients in eligible trials are equally likely to be randomized to any of the interventions. The transitivity assumption is required for valid indirect comparisons and will be further explored by examining the distribution of potential effect-modifiers across treatment comparisons (Higgins et al 2019).

In addition, we will also explore whether differences across the sham interventions and other control groups could cast doubts in the transitivity assumption.

A common between-study variance (τ2) will be assumed across treatment comparisons within a network (Higgins et al 2019). Heterogeneity will be quantified by comparing the τ2 with its empirical distributions (Rhodes et al 2015) and the magnitude will be classified into low, moderate and high.

The agreement between direct and indirect evidence will be evaluated within closed loops with the separating indirect from direct evidence approach and in the entire network with a design-by-treatment interaction test (Higgins et al 2019). Tests of incoherence have low statistical power, and thus, sources of incoherence will be explored even in the absence of statistical significance.

Small-study effects and the potentially publication bias will be examined for the primary outcome and dropouts due to any reason with contour-enhanced funnel plots for pairwise meta-analysis when more than 10 studies are available (Higgins et al 2019), and comparison-adjusted funnel plots assuming the direction of bias towards newer interventions (Chaimani et al 2013). We will further evaluate reporting bias for the entire networks using the RoB-MEN tool.

The confidence in the evidence will be evaluated for the primary outcome and dropouts due to any reason using the CINeMA approach (Nikolakopoulou et al 2020).

Data analysis will be conducted with the package netmeta in R statistical software. Alpha will be set at two-sided 5%, except for heterogeneity and incoherence tests at 10%.

Analysis of subgroups or subsets

We will investigate potential sources of heterogeneity and/or incoherence in the primary outcome with subgroup analyses of a) baseline severity of overall symptoms, b) definition of treatment-resistance, c) publication year, d) sample size, and e) treatment duration. We will also conduct a subgroup analysis to explore differences in the pre-post change in overall symptoms among different sham interventions and other control groups.

Sensitivity analyses

The robustness of the results for the primary outcome will be investigated with sensitivity analysis by excluding studies a) that were single-blind, b) with an overall high risk of bias, c) with implied randomization, d) that did not use

operationalised criteria, e) in which patients were assumed to receive antipsychotics, f) that required all patients to have treatment-resistant positive symptom domains, g) with rating scales of positive symptoms, h) imputed values, and i) from mainland China as well as by defining j) different nodes for active and inactive sham interventions and k) different nodes for specific NIBS protocols.

Contact details for further information

Carolin Lorenz

carolin.lorenz@tum.de

Organisational affiliation of the review

Department of Psychiatry and Psychotherapy, School of Medicine, Technical University of Munich, Munich,

Germany

https://www.tum.de/

Review team members and their organisational affiliations

Ms Carolin Lorenz. Department of Psychiatry and Psychotherapy, School of Medicine, Technical University of Munich, Munich, Germany

Dr Spyridon Siafis. Department of Psychiatry and Psychotherapy, School of Medicine, Technical University of Munich, Munich, Germany

Mrs Hui Wu. Department of Psychiatry and Psychotherapy, School of Medicine, Technical University of Munich, Munich, Germany

Dr Johannes Schneider-Thoma. Department of Psychiatry and Psychotherapy, School of Medicine, Technical University of Munich, Munich, Germany

Dr Irene Bighelli. Department of Psychiatry and Psychotherapy, School of Medicine, Technical University of Munich, Munich, Germany

Yikang Zhu. Shanghai Key Laboratory of Psychotic Disorders, Shanghai Mental Health Centre, Shanghai Jiao Tong University School of Medicine, China

Chunbo Li. Shanghai Key Laboratory of Psychotic Disorders, Shanghai Mental Health Centre, Shanghai Jiao Tong University School of Medicine, China and Institute of Psychology and Behavioral Science, Shanghai Jiao Tong University, China;

Mr Wulf-Peter Hansen. BASTA-Das Bündnis für psychisch erkrankte Menschen, Munich, Germany

Professor Frank Padberg. Department of Psychiatry and Psychotherapy, LMU Klinikum, Munich, Germany

Professor Georgia Salanti. Institute of Social and Preventive Medicine (ISPM), University of Bern, Bern, Switzerland

Professor Stefan Leucht. Department of Psychiatry and Psychotherapy, School of Medicine, Technical University of Munich, Munich, Germany

Collaborators

Dr Farhad Shokraneh. King's Technology Evaluation Centre, King’s College London, London, UK

Type and method of review

Systematic review

Anticipated or actual start date

01 March 2023

Anticipated completion date

30 November 2023

Funding sources/sponsors

German Federal Ministry of Education and Research (Bundesmininsterium für Forschung und Bildung/BMBF)

Grant number(s)

State the funder, grant or award number and the date of award

Grant number 01KG2206

Conflicts of interest

In the last 3 years, Stefan Leucht has received honoraria for consulting or lectures from LB Pharma, Lundbeck, Otsuka, TEVA, Lohmann, Geodon Richter, Recordati, Boehringer Ing., Sandoz, Janssen, Lilly, SanofiAventis, Servier and Sunovion. Others have no conflicts of interest to declare.

Yes

Language

English

Country

China, England, Germany, Switzerland

Stage of review

Review Ongoing

Subject index terms status

Subject indexing assigned by CRD

Subject index terms

Brain; Humans; Meta-Analysis as Topic; Schizophrenia; Stereotaxic Techniques; Systematic Reviews as Topic; Transcranial Direct Current Stimulation; Transcranial Magnetic Stimulation

Date of registration in PROSPERO

03 April 2023

Date of first submission

23 March 2023

Stage of review at time of this submission

Stage	Started	Completed
Preliminary searches	Yes	No
Piloting of the study selection process	Yes	No
Formal screening of search results against eligibility criteria	No	No
Data extraction	No	No
Risk of bias (quality) assessment	No	No
Data analysis	No	No

The record owner confirms that the information they have supplied for this submission is accurate and complete and they understand that deliberate provision of inaccurate information or omission of data may be construed as scientific misconduct.

The record owner confirms that they will update the status of the review when it is completed and will add publication details in due course.

Versions

03 April 2023

03 April 2023
